# N-terminal gelsolin fragment potentiates TRAIL mediated death in resistant hepatoma cells

**DOI:** 10.1038/s41598-017-13131-7

**Published:** 2017-10-09

**Authors:** Keith Meyer, Young-Chan Kwon, Ratna B. Ray, Ranjit Ray

**Affiliations:** 1 Departments of Internal Medicine and Pathology, Saint Louis University, Missouri, USA; 2 Pathology, Saint Louis University, Missouri, USA

## Abstract

TNF-α related apoptosis-inducing ligand (TRAIL) selectively kills tumor cells, without damaging normal cells. TRAIL receptors facilitate induction of apoptosis for selective elimination of malignant cells. However, some cancer cells have developed resistances to TRAIL which limits anticancer potential. Gelsolin, a multifunctional actin-binding protein, mediates cell death involving the TRAIL receptors in the hepatic stellate cell line, LX2. Here, we have shown that conditioned medium (CM) containing gelsolin fragments or an N-terminal gelsolin fragment (amino acid residues 1–70) in the presence of TRAIL impairs cell viability of TRAIL resistant transformed human hepatocytes (HepG2). Cell growth regulation by CM and TRAIL was associated with the modulation of p53/Mdm2, Erk and Akt phosphorylation status. The use of N-terminal gelsolin peptide_1–70_ alone or in combination with TRAIL, induced inhibition of Akt phosphorylation and key survival factors, Mdm2 and Survivin. Treatment of cells with an Akt activator SC79 or p53 siRNA reduced the effects of the N-terminal gelsolin fragment and TRAIL. Together, our study suggests that the N-terminal gelsolin fragment enhances TRAIL-induced loss of cell viability by inhibiting phosphorylation of Akt and promoting p53 function, effecting cell survival.

## Introduction

Hepatocellular carcinoma (HCC) is a malignancy of worldwide significance and has become increasingly important in the United States. Novel pharmacological modality is urgently needed for HCC treatment. TRAIL may be of potential use as an anticancer drug for tumor selectivity, minimal side effect in animal models, and promising results from phase I/II clinical studies^1^. TRAIL initiated intracellular apoptosis signal transduction involves the TRAIL-death receptors (DR4 and DR5), Fas-associated protein with death domain (FADD) and caspase signaling^2^. TRAIL can activate the extrinsic pathway of cell death by binding to the death receptors, DR4 and DR5. The apoptosis signal of TRAIL may be amplified by mitochondria, which is regulated by members of the Bcl-2 family. However, HCC cells exhibit a major resistance to TRAIL-induced cell death.

Due to varying factors within individual established tumors leading to resistance to TRAIL mediated growth inhibition, the antitumor effect of TRAIL as a single agent is limited. Cytotoxic drugs, such as doxorubicin, methotrexate and others induce apoptosis along with TRAIL^3^. Several mechanisms work for cytotoxic drugs sensitizing tumor cells for TRAIL-induced apoptosis. Among them, p53 is activated in tumor cells by several cytotoxic drugs and mediates gene regulation, apoptosis and cell cycle arrest. Several proteins mediate TRAIL-induced apoptosis, including TRAIL receptor 2 or DR5 as p53 target gene. Therefore p53-mediated gene regulation is a mechanism for mediating apoptosis of cytotoxic drugs and TRAIL^4^. Activation of the PI3K/Akt pathway is associated with tumorigenesis and resistance to apoptosis, and inhibition of Akt activation also enhances TRAIL mediated cell death^5–7^.

Our previous study suggested that conditioned medium (CM) from immortalized human hepatocytes (IHH) induced apoptosis in human hepatic stellate cells (LX2). Peptide mass fingerprinting of a purified soluble mediator from CM indicated that gelsolin fragments may play a role in LX2 apoptosis^8^, and similarly modulated MAPK/Akt/Mdm2/Bcl2, and enhanced Bax, in the absence of TRAIL (unpublished observations). Further studies indicated that the N-terminal gelsolin_1–70_ fragment also induces LX2 cell death in the absence of TRAIL and decreases Bcl2 expression. Gelsolin, a multifunctional actin-binding protein, is downregulated in several types of tumors and its abnormal expression is one of the most common defects noted in invasive breast carcinoma^9^. Loss of gelsolin, a tumor suppressor, is one of the most frequently occurring molecular defects in breast cancers of diverse etiologies in human, mouse, and rat^10^. CM increased the expression of TRAIL receptors on LX2 surface, and induced apoptosis by a caspase dependent mechanism^11^. Gelsolin is secreted from several mammalian cell types. Originally defined by its interactions with actin, plasma gelsolin circulates in mammalian blood at concentrations of 200–300 µg/ml^12–15^. An earlier study identified an N-terminal gelsolin fragment obtained by caspase 3 mediated cleavage in response to IFN-γ and TNF-α exposure^16^. This fragment reduced cell viability in a manner similar to our previous work^8,11^. Further analysis determined that this activity was restricted to a region encompassing amino acids 1–70 in the gelsolin sequence^11^, and antibody against a linear B-cell epitope from this region inhibits stellate cell death (unpublished observation). This fragment upregulated TRAIL-R1/TRAIL-R2, and involved caspase 3 activation. The apoptotic activity of the N-terminal gelsolin fragment was restricted to activated, not quiescent, stellate cells indicating its potential application as an anti-fibrotic agent.

Sorafenib, a multikinase inhibitor, improves overall survival in patients with advanced HCC^17^. However, there is urgent need for additional pharmacological modalities for HCC. Gelsolin has a tumor suppressor activity in breast cancers^9,16^, although the role of gelsolin in HCC remains unknown. Here, we examined whether gelsolin can potentiate TRAIL mediated cell death in resistant human hepatoma cells. Our findings indicated that the gelsolin fragment sensitizes transformed hepatocytes to TRAIL-mediated apoptosis through modulation of cell survival pathways. The results suggested that the combination of TRAIL and the N-terminal gelsolin fragment may be effective for treatment of complexities of HCC associated desmoplasia.

## Results

### Conditioned medium from immortalized hepatocytes potentiates TRAIL mediated cell death in transformed cells

**Figure 1:**
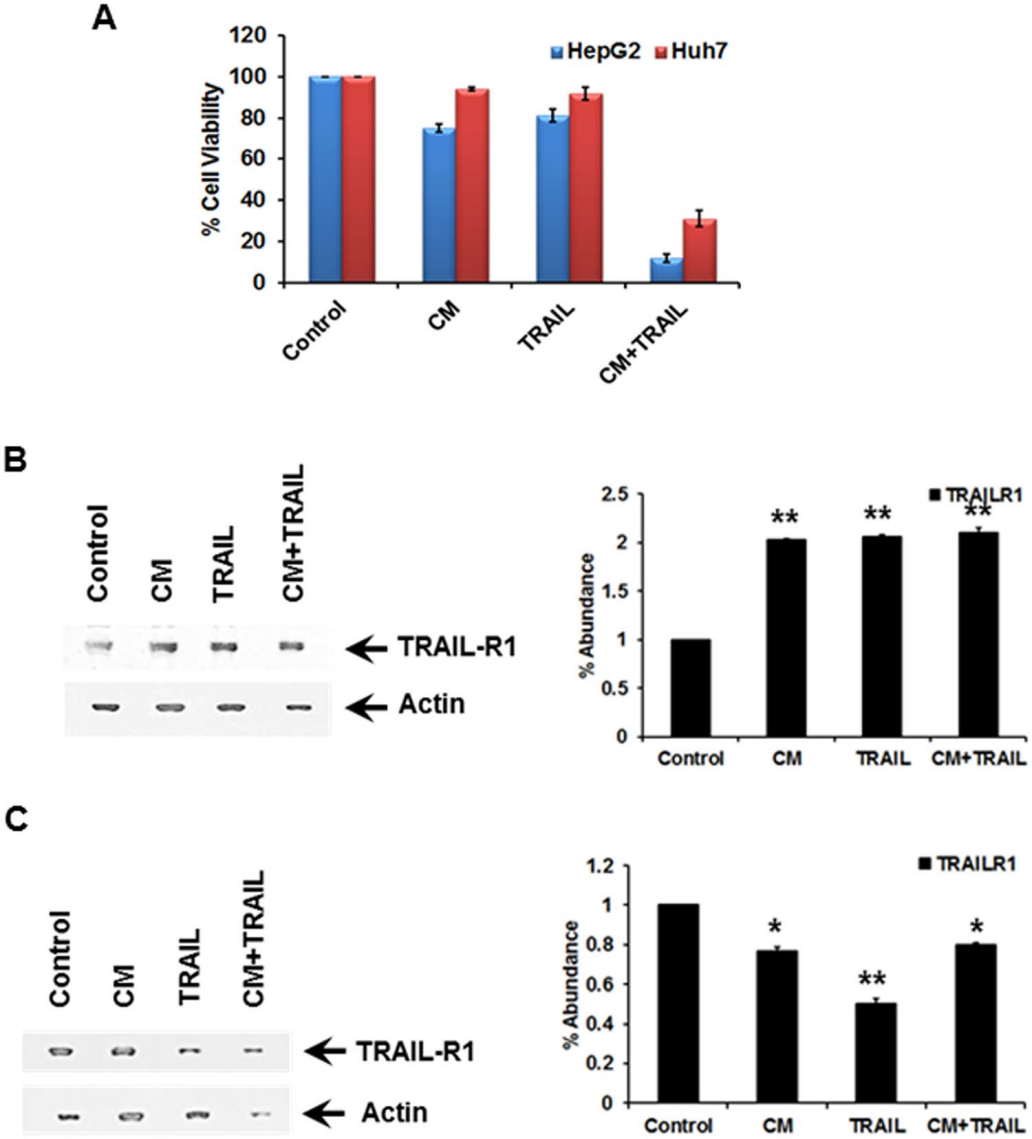
Treatment of TRAIL insensitive transformed hepatic cells with CM and/or TRAIL enhances death sensitivity. HepG2 and Huh7 cells (panel A) were treated for 24 hours with CM as a source of gelsolin fragment, TRAIL (50 μg/ml and 100 μg/ml, respectively) or both CM and TRAIL. TRAIL-R1 receptor expression was analyzed in HepG2 (panel B) and Huh7 (panel C) cells by Western blot. Note that cropped gel images are used and the gels were run under the same experimental conditions. Densitometric scanning of the Western blot results are shown at the bottom of respective panels. Values represent mean from three independent experiments ± SD and are shown as error bars. Statistical significance was analyzed using the two-tailed Student’s t-test: *P < 0.05, **P < 0.01.

Normal human hepatocytes do not express detectable TRAIL receptor 1 (TRAIL-R1), and only a limited level of receptor 2 (TRAIL-R2)^18^. To evaluate the mechanisms of tumor resistance to TRAIL and the ability of gelsolin to sensitize cells to TRAIL mediated cell death, we investigated human hepatoma (HepG2) and hepatocellular carcinoma (Huh7) cells exhibiting reduced sensitivity to TRAIL^19^. Previous studies have established reduced sensitivity of HepG2 and Huh7 cells to TRAIL^20–24^ and subsequent analysis in our laboratory concurred with these results (data not shown) and allowed for selection of the TRAIL dose used in each cell line. Treatment of the cells with CM from IHH or TRAIL (25ng/ml for HepG2 and 50ng/ml for Huh7) alone induced modest (10–20%) cell death (Fig. 1A). However, a combination of both CM and TRAIL reduced cell viability in HepG2 (~90%) and Huh7 (~70%) cells, indicating that CM sensitizes resistant hepatocytes to TRAIL mediated death.

We analyzed the expression of TRAIL-R1 in HepG2 and Huh7 (Fig. 1B,C) cells. A modest increase in TRAIL-R1 was observed in HepG2 cells after treatment with CM/TRAIL. On the other hand, Huh7 cells did not display an increase in TRAIL receptor upon treatment with CM, and had a similar high expression level in the untreated control. These results suggest that the expression of TRAIL receptor differs between these two cell lines, and the induction of cell death by the combination of CM and TRAIL may not be identical. Further, a decrease in TRAIL receptor expression in treated Huh7 cells may indicate that modulation of this receptor does not relate to TRAIL mediated cell death. We further evaluated the effects CM and the N-terminal gelsolin_1–70_ fragment upon cell survival pathways independent of TRAIL receptor enhancement as described below.

### Combination of CM and TRAIL impairs Akt, Erk, or MAPK activation in transformed cells

**Figure 2:**
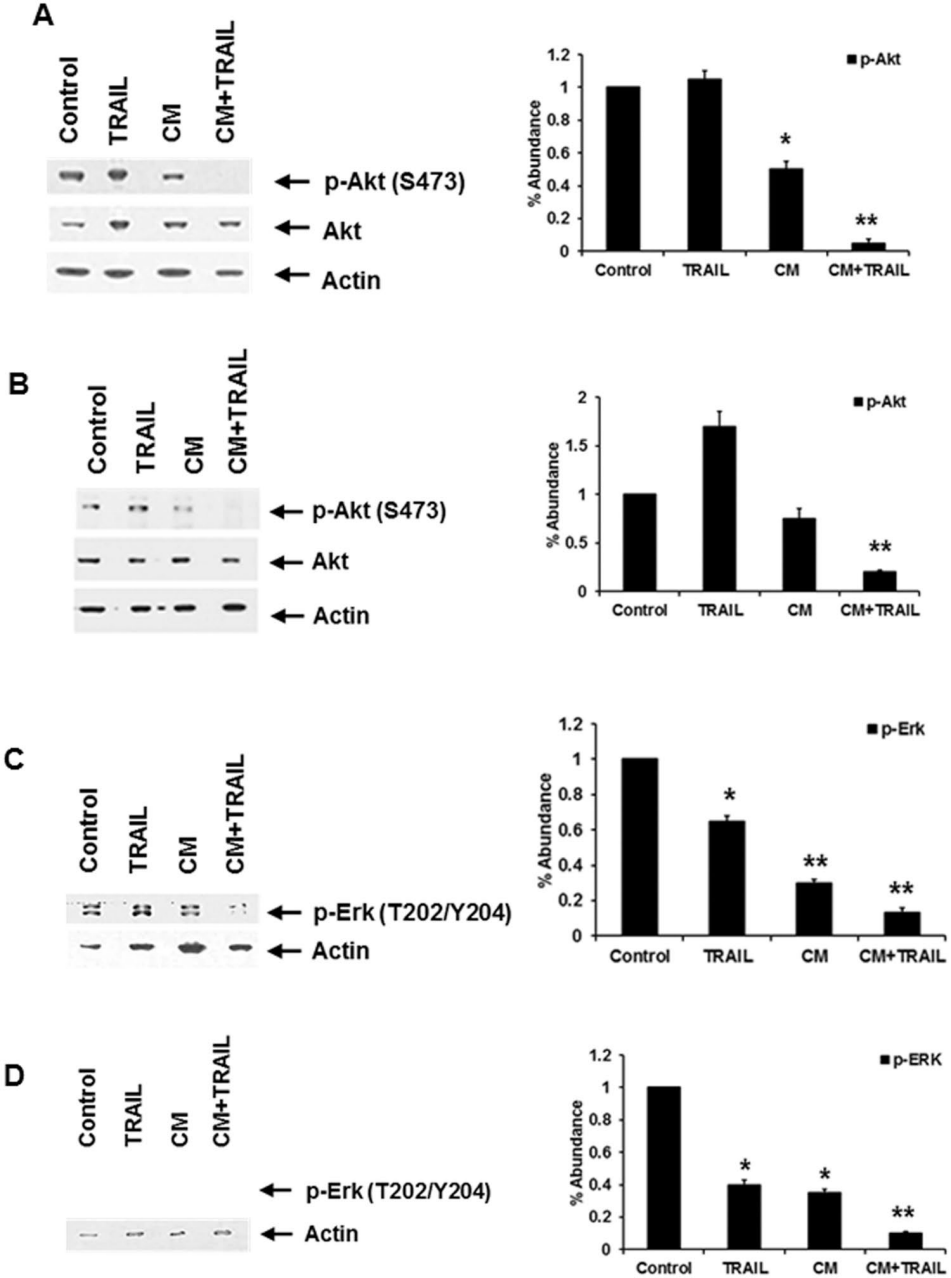
CM in association with TRAIL inhibits Akt and Erk activation. Akt and Erk activation was analyzed in HepG2 (panels A and C) and Huh7 (panels B and D) cells. CM was added on cells 24 hours prior to the addition of TRAIL. Densitometric scan data is provided. Each panel is representative of three independent experiments. Note that cropped gel images are used and the gels were run under the same experimental conditions.

The activation of the phosphoinositide 3-kinase (PI 3-kinase)-Akt signaling cascade protects cells from apoptosis^25^. In addition, the triggering of stress-activated kinases, concomitant with the inhibition of the extracellular signal-regulated kinase (Erk) pathway, are observed in cells undergoing programmed cell death. The PI3K and MAPK pathways are associated with cell survival in response to TRAIL mediated apoptosis. Treatment with CM reduced levels of Akt phosphorylated at Ser473 in both HepG2 and Huh7 cells (Fig. 2A,B **)**. However, the levels of Akt remained constant upon treatment of HepG2 and Huh7 cells with TRAIL. Further, co-treatment with CM and TRAIL led to greatly inhibit Akt activation. Next, we analyzed expression of Erk in response to TRAIL, CM, and a combination of both. The use of TRAIL individually exhibited a moderate effect on the activation of Erk, while Erk activation was decreased in CM treated cells (Fig. 2C,D). However, the use of both in concert led to a significant decrease in the phosphorylation of Erk protein, with the onset of cell death. Modulation of both Akt and Erk activation may contribute to the loss of TRAIL resistance.

### CM in the presence of TRAIL modulates p53/Mdm2 activation in transformed hepatocytes

**Figure 3:**
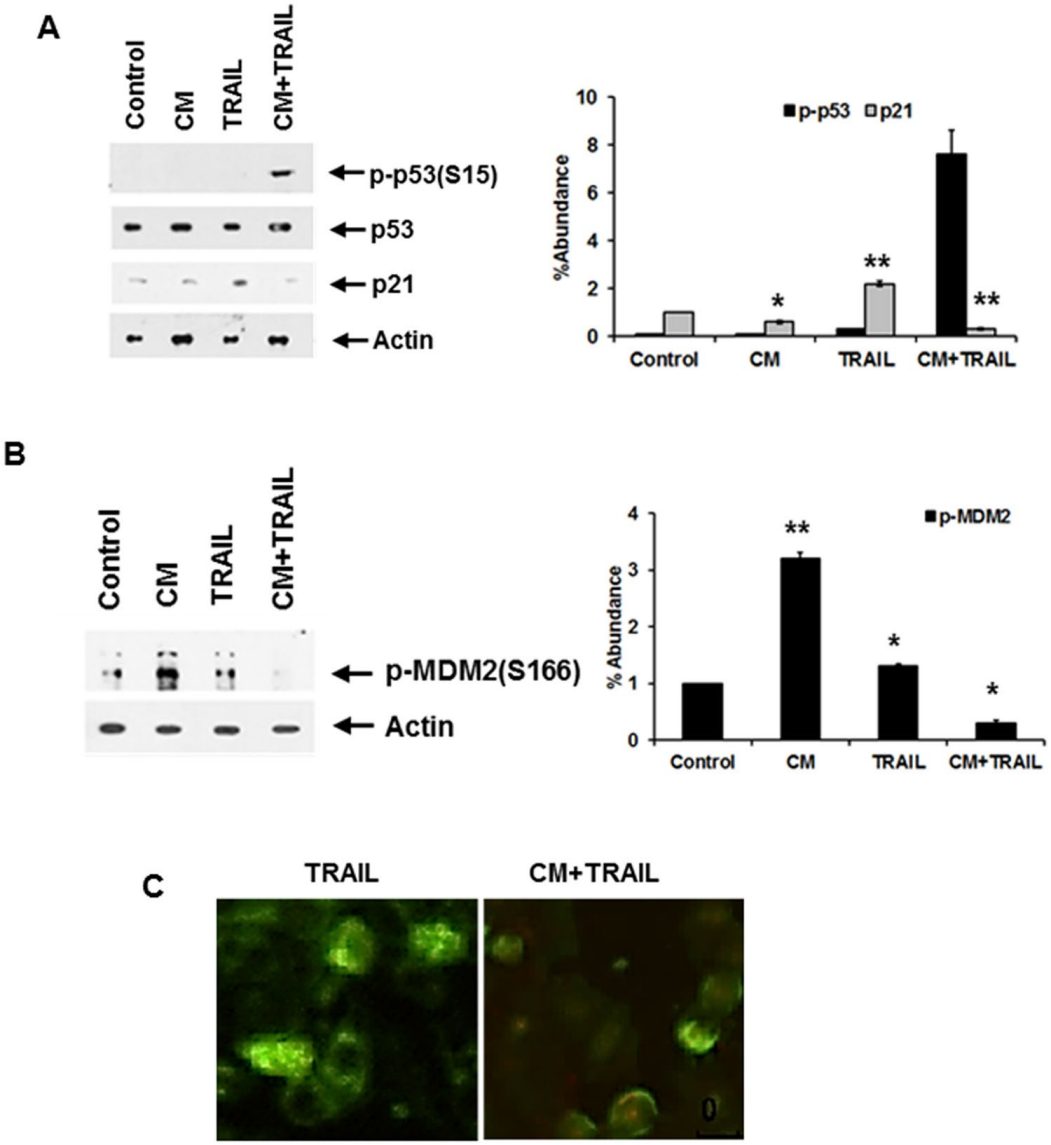
CM and TRAIL reduces association between p53 and Mdm2. p53 and Mdm2 phosphorylation was analyzed in CM, TRAIL, and CM + TRAIL treated HepG2 cells (panels A and B). Cellular phosphorylation status of p53 at Serine15 residue was apparent when cells were exposed to both CM and TRAIL, but total p53 levels were not affected. Phosphorylated Mdm2 was reduced in the presence of both CM and TRAIL (panel B). Densitometric scan data is provided. Note that cropped gel images are used and the gels were run under the same experimental conditions. Subcellular localization of p53 and Mdm2 is shown by immunofluorescence (panel C). Co-localization of p53 and Mdm2 was noted in HepG2 control cells, and their association noted in the presence of TRAIL. This interaction was not observed in cells treated with CM and TRAIL when examining multiple fields. Each panel is representative of three independent experiments.

Since p53 and Akt are major but opposing players in signaling pathways that determine cell survival, it is conceivable that there may be cross-talk between these two proteins to enable integration of signaling information towards death or survival. HepG2 cells carry wild-type p53, whereas Huh7 cells have point mutations in p53 at codon 220 resulting in substitution from tyrosine to cysteine to inhibit functional activity^26^. We analyzed the potential for CM/TRAIL to affect p53 activation status. Phosphorylation of p53 on the Serine15 residue was apparent when HepG2 cells were treated with both CM from IHH and TRAIL, without an increase in total p53 content (Fig. 3A). In similar fashion, p53 phosphorylation was also noted in the p53 defective Huh7 cell line, indicating that the associated phosphorylation was conserved even in the absence of p53 functionality (data not shown). p53 is the main transcriptional regulator of p21, and p21 has been observed to inhibit TRAIL mediated apoptosis^27^. p21 levels were increased in HepG2 cells upon TRAIL exposure, but this effect was reduced in the presence of IHH CM. Modulation of p53 activation in HepG2 cells may, in part, contribute to gelsolin and TRAIL mediated cell death, and may partly explain decreased cell death apparent in Huh7 cells as compared to HepG2. However, a p53 negative hepatoma cell line, Hep3B, exhibited significant cell death in the presence of conditioned medium alone (data not shown). This may further indicate that there is more than one cellular pathway affected by CM leading to cell death.

The major physiological regulator of p53 function is the Mdm2 oncoprotein. Mdm2 through its action as a p53-specifc E3 ubiquitin ligase ubiquitinates p53 and targets the protein for rapid proteasomal degradation^28–30^. When Akt becomes active, phosphorylation occurs at an Akt consensus site (Serine166) within the Mdm2 protein modulating cellular interactions which promote p53 degradation^31^. Our, results suggested that a combination of CM and TRAIL led to a decrease in activated Akt (shown previously in Fig. 2A,B). Densitometric scanning for comparison of protein expression levels are shown at the bottom of each panel. Therefore, Akt may modulate the p53 pathway by altering the intracellular translocation of Mdm2^31^. Consequently, we examined the phosphorylation of Mdm2 at Ser166. The treatment of both HepG2 and Huh7 cells with CM and TRAIL displayed a much reduced level of activated Mdm2 (Fig. 3B and data not shown). Phosphorylations of Mdm2 at Serine166 and p53 at Serine15 are associated with cellular interactions between these two proteins, leading to modulation of p53 activity. Control cells displayed significant association between p53 and Mdm2 when exposed to TRAIL alone (Fig. 3C). In contrast, the association between p53 and Mdm2 was not apparent in cells treated with both CM and TRAIL. These results indicate that p53 functional activity may contribute to TRAIL mediated cell death in the presence of gelsolin, but is not the only mechanism by which decreased cell viability may occur via these ligands.

### Combination of CM and TRAIL decreases survivin and Bcl2 in transformed hepatocytes

**Figure 4:**
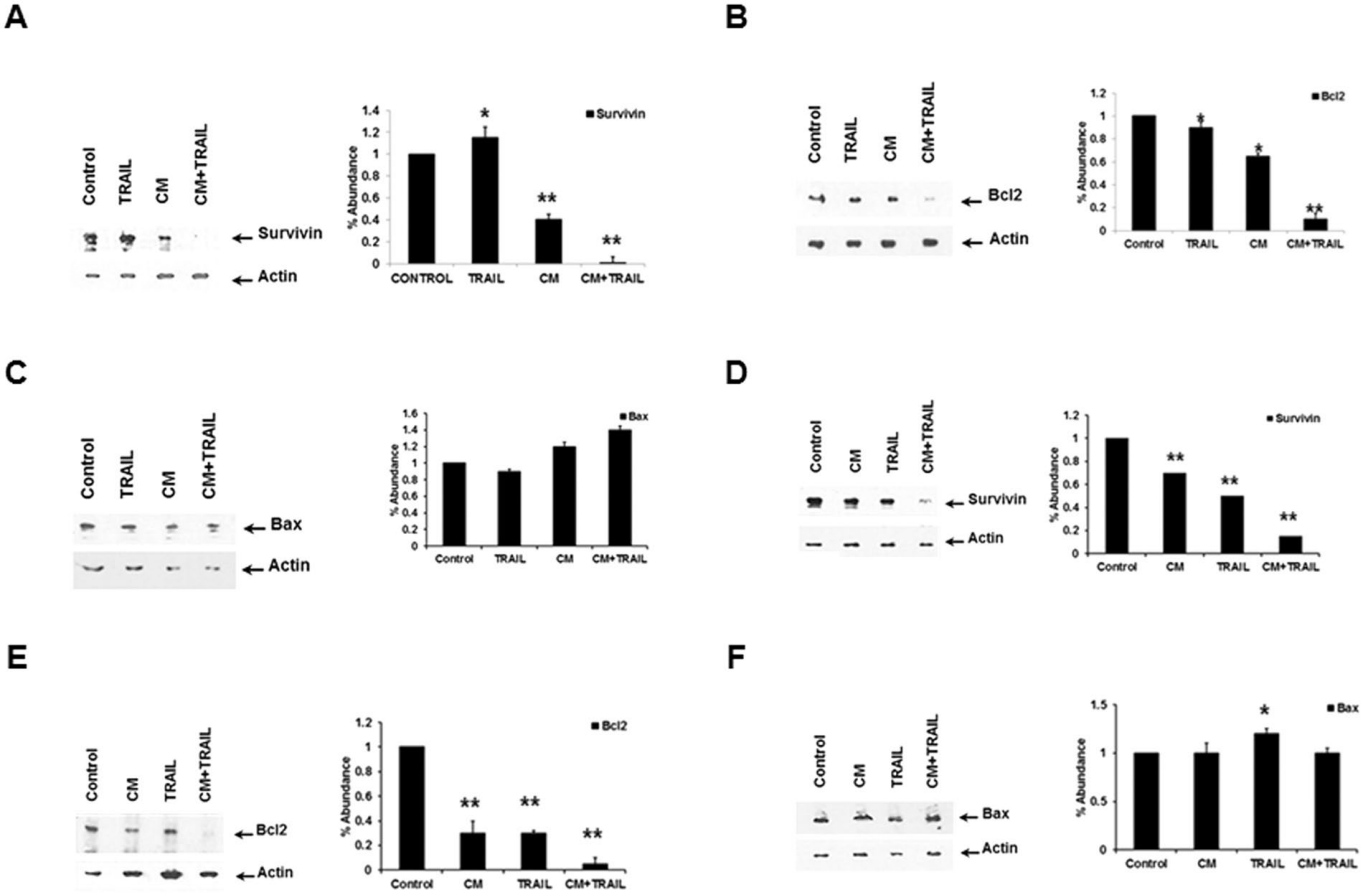
CM and TRAIL reduce cell survival associated proteins. Analysis of survivin and the BH3 proteins (Bcl2 and Bax) in cells treated with CM, TRAIL, and CM + TRAIL. HepG2 cells exhibited a decrease in survivin and Bcl2 (panels A and B), while Bax protein level remained unchanged (panel C). In a similar experiment, Huh7 exhibited decreased survivin and Bcl2 expression when treated with both CM + TRAIL (panels D and E), while Bax level increased (panel F). Densitometric scan data is provided. Note that cropped gel images are used and the gels were run under the same experimental conditions. Each panel is representative of three independent experiments.

The downregulation of survivin or Bcl-2 by siRNA sensitizes resistant melanoma cells to TRAIL-induced apoptosis^32^. Here, we analyzed the ability of a combination of IHH CM and TRAIL to modulate the expression of survivin and Bcl-2, as well as the pro-apoptotic BH3 family member, Bax. The expression of survivin or Bcl-2 was decreased in HepG2 cells treated with both CM and TRAIL, with very little change in the expression level of Bax (Fig. 4A–C). Densitometric scanning for comparison of protein expression levels are shown at the bottom of each panel. Further, treatment of Huh7 cells with either CM or TRAIL exhibited a modest change in each of these proteins (Fig. 4D–F). In contrast, the use of both ligands displayed a significant decrease in both survivin and Bcl-2 expression (Fig. 4D,E), but did not affect the level of Bax expression in Huh7 cells (Fig. 4C).

### Treatment of cells with N-terminal gelsolin fragment decreases HCC cell proliferation

**Figure 5:**
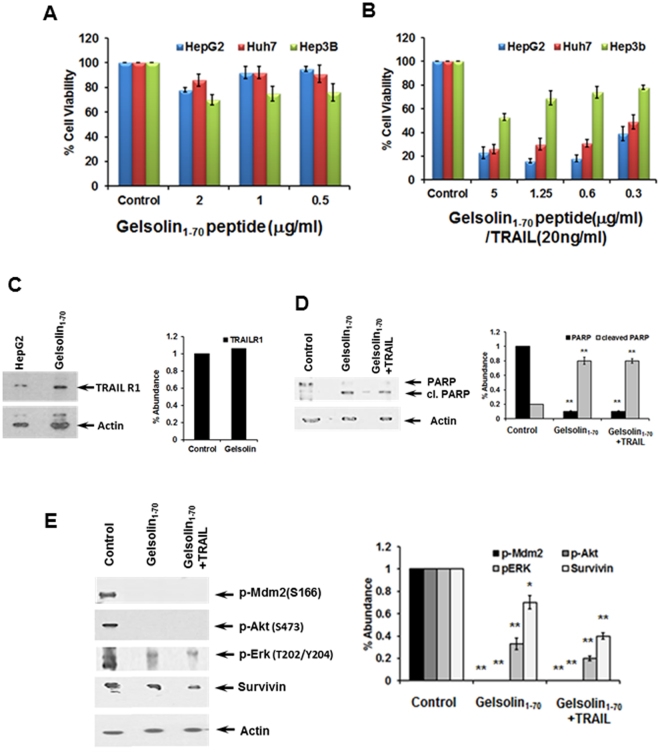
N-terminal gelsolin_1–70_ fragment in combination with TRAIL impairs transformed hepatocyte growth. Treatment of HepG2, Huh7 and Hep3B with an N-terminal gelsolin fragment (panel A) displayed a poor or modest reduction in cell viability by MTT assay. On the other hand, cells treated with different concentrations of the gelsolin_1–70_ peptide together with TRAIL displayed significant loss of cell viability after 72 hours, while Hep3B cells displayed only a slight increase in cell death over cells treated with the gelsolin fragment alone (panel B), and TRAIL R1 modulation by gelsolin_1–70_ (panel C). Western blot analysis for PARP cleavage in HepG2 cells and densitometric scanning results are shown (panel D). Cell survival proteins in HepG2 cells treated with gelsolin_1–70_ with or without TRAIL were analyzed separately (panel E). Western blot analyses for activated forms of cell regulatory (p-Mdm2, p-Akt473, p-ERK) and survivin proteins, and densitometric scan results are shown. Each panel is representative of three independent experiments and mean values ± SD are shown as error bars. Note that cropped gel images are used and the gels were run under the same experimental conditions.

We examined HepG2, Huh7 and Hep3B cell lines following treatment with N-terminal gelsolin peptide_1–70_. Each cell line exhibited minimal loss in cell viability, with the most significant level (~30%) exhibited by Hep3B (Fig. 5A). Further analysis was undertaken to determine if a reduction in cell viability mirrored that seen with CM and TRAIL treatment in the transformed hepatocyte cell lines in the presence of the gelsolin_1–70_ peptide and TRAIL. Cells were treated with a fixed concentration of TRAIL and varied concentrations of the gelsolin_1–70_ peptide (0.3–5.0 ug/ml). A significant reduction in cell viability (>70%) was observed in HepG2 and Huh7 cells (Fig. 5B). However, a p53 null cell line, Hep3B, exhibited minimal effect with the addition of TRAIL only at the highest concentration of the gelsolin fragment used in this assay. TRAILR1 modulation for HepG2 and Huh7 was similar to that seen using CM/TRAIL (Fig. 5C). PARP cleavage to a 89 Kd fragment is a marker for apoptotic cell death. Western blot analysis for cell death associated protein PARP/cleaved fragment in HepG2 and densitometric scanning results are shown (Fig. 5D).

We analyzed selected proteins to identify key targets for the gelsolin fragment leading to TRAIL sensitization of HepG2 cells as a representative cell line. Phosphorylated Mdm2, Akt and Erk were significantly reduced in CM or gelsolin_1–70_ treated cells (Fig. 5E). Further, survivin levels were also reduced in the presence of CM or gelsolin_1–70_/TRAIL. Densitometric scanning for comparison of protein expression levels are shown at the bottom of each panel. These results indicate further that the gelsolin_1–70_ fragment exerts significant influence upon transformed cells, which are resistant to TRAIL potentiating cell death.

### Activation of Akt by sc-79 enhanced HepG2 cell viability to treatment with the N terminal gelsolin fragment and TRAIL

**Figure 6:**
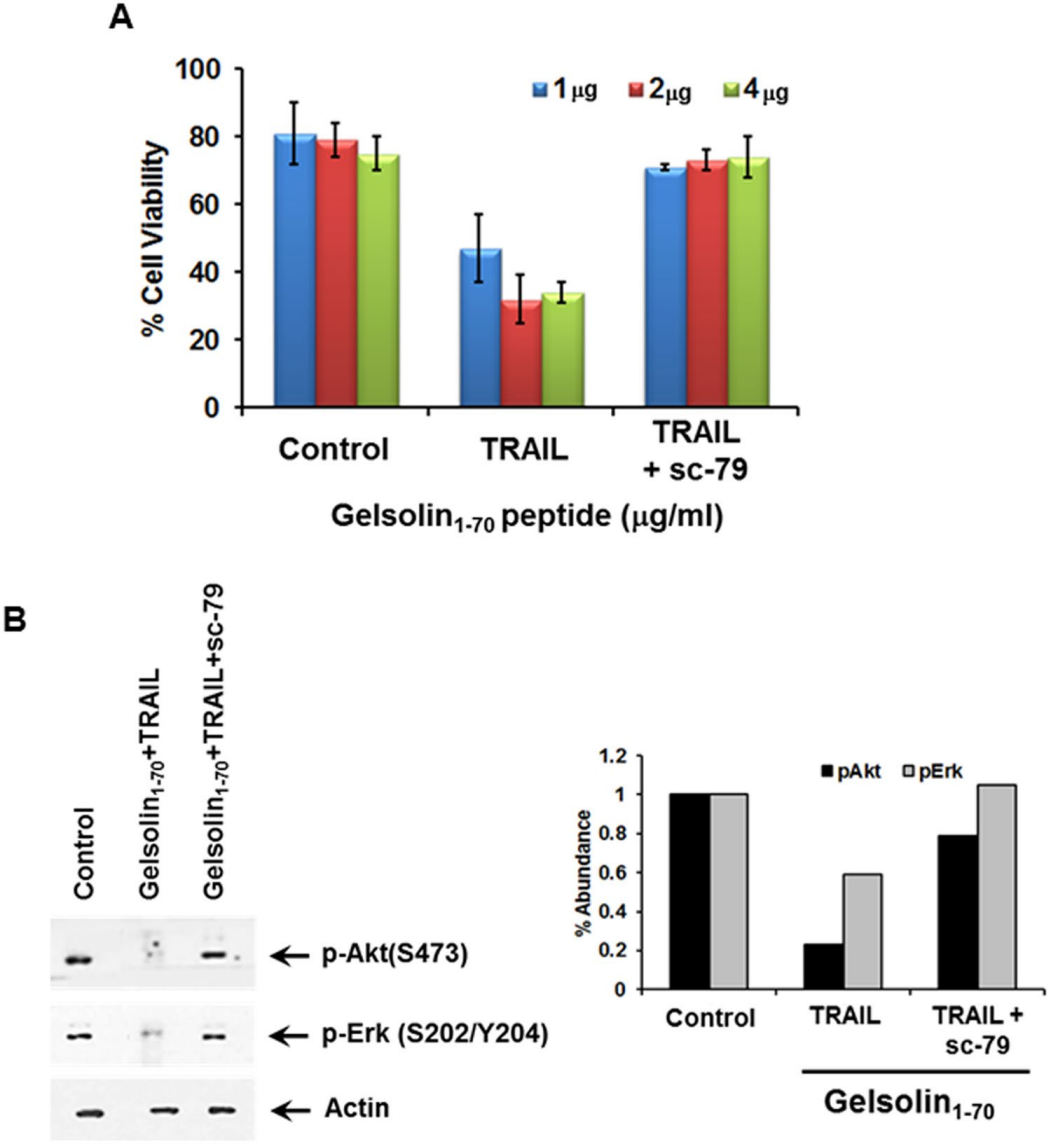
Treatment of HepG2 with an activator of Akt (sc-79) decreased the loss in cell viability in cells treated with an N-terminal gelsolin_1–70_ fragment and TRAIL. Treatment of cells with N-terminal gelsolin_1–70_ fragment and TRAIL in the presence of an Akt activator enhances cell viability at multiple doses of the fragment (1, 2, and 4 μg/ml) after 48 hours incubation by MTT assay (panel A). Western blot analysis exhibited an increase in the phosphorylation of Akt and Erk in sc-79 treated cells (panel B). Densitometric scanning results are shown on the right. Each panel is representative of three independent experiments and mean values ± SD are shown as error bars. Note that cropped gel images are used and the gels were run under the same experimental conditions.

To activate the Akt pathway in the presence of gelsolin_1–70_/TRAIL, we used the Akt activating agent; Sc-79, which specifically binds to the PH domain of Akt, Sc-79-bound Akt adopts a conformation favorable for phosphorylation by upstream protein kinases^33^. Activation of the Akt pathway is associated with a decrease in sensitivity to TRAIL treatment^34^. As above, treatment with gelsolin_1–70_/TRAIL led to a decrease in HepG2 cell viability, as compared to untreated cells and those treated with gelsolin peptide alone. The addition of SC79 in cells treated with gelsolin_1–70_/TRAIL restored cell viability (Fig. 6A). Expression of activated Akt in the presence of sc-79 and gelsolin_1–70_/TRAIL are shown by Western blot analysis and densitometric scanning (Fig. 6B). Interestingly, the expression of activated Erk was also observed after this treatment. This data indicates that the loss of activated Akt may play an important role in the observed decrease in cell viability which TRAIL treated HepG2 cells undergo in the presence of the gelsolin fragment.

### Inhibition of p53 expression by siRNA enhanced cell viability in p53 positive HepG2 cells treated with the gelsolin fragment and TRAIL

**Figure 7:**
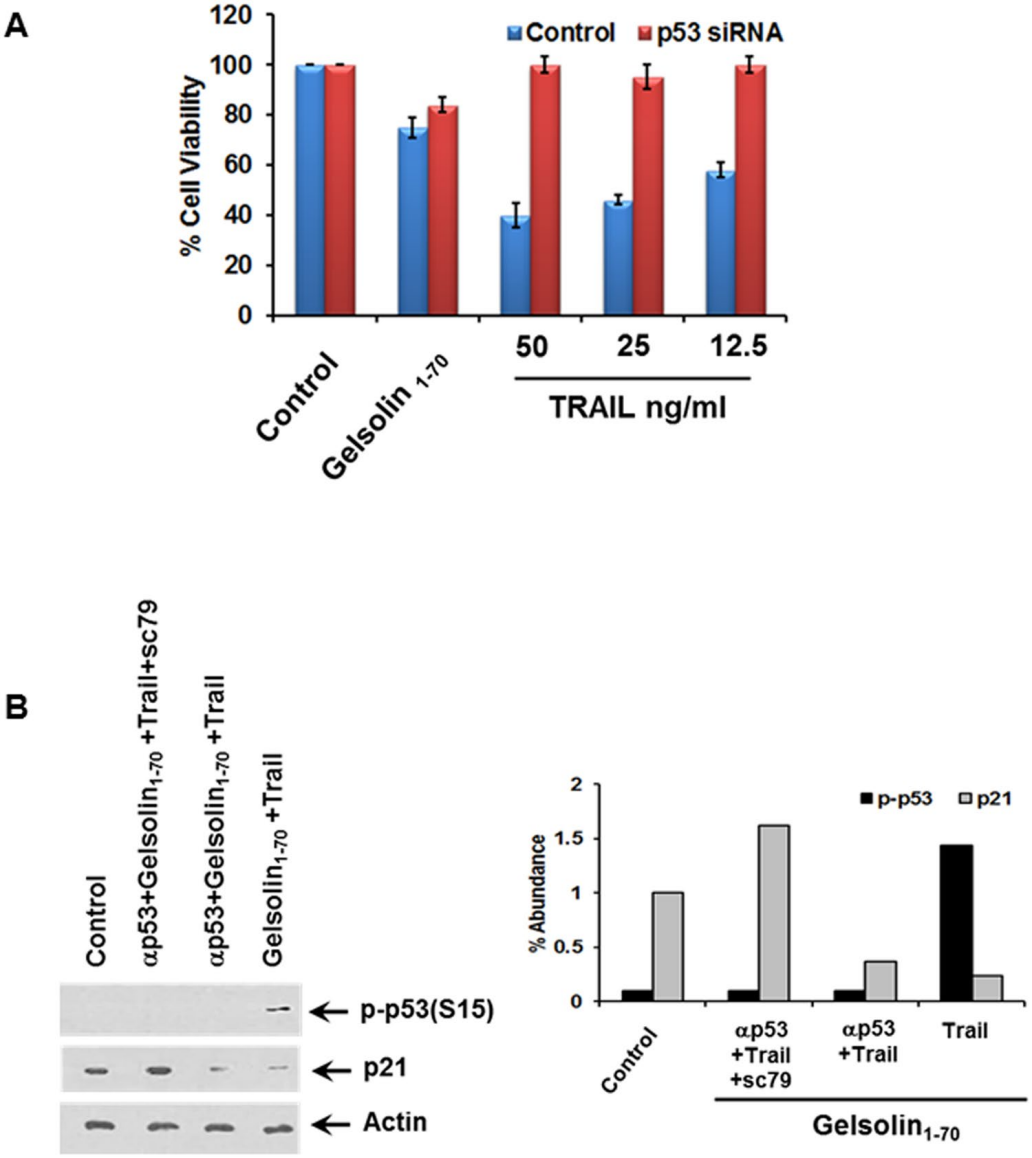
Inhibition of p53 expression decreased the loss in cell viability in p53 positive cells treated with an N-terminal gelsolin_1–70_ fragment and TRAIL. Treatment of HepG2 cells with siRNA to p53 prior to exposure to the N-terminal gelsolin_1–70_ fragment and TRAIL enhanced cell viability after 48 hours of treatment (panel A). Treatment of HepG2 cells with siRNA to p53 prior to exposure to the N-terminal gelsolin_1–70_ fragment and TRAIL inhibited phosphorylation of p53 at S15 and enhanced p21 expression by Western blot analysis (panel B). Densitometric scanning results are shown on the right. Each panel is representative of three independent experiments and mean values ± SD are shown as error bars. Note that cropped gel images are used and the gels were run under the same experimental conditions.

As the p53 null cell line; Hep3b, displayed minimal effect by the co-treatment of the gelsolin fragment and TRAIL, we further analyzed the contribution p53 makes in mediating this loss in cell viability. Cells treated with gelsolin_1–70_ fragment displayed increased viability when prior treated with a siRNA to p53 (Fig. 7A), even at increased concentration of TRAIL. Analysis of protein expression by Western blot indicated that not only was p53 expression eliminated, but the phosphorylation of p53 at Ser15 was not present (Fig. 7B). Interestingly, the addition of the sc-79 as an Akt activating agent led to an increase in p21 expression in cells treated with p53 siRNA. Similarly, a constitutively active form of Akt has previously been noted to induce p21 expression in human endothelial cells^35^. Thus, the role of p53 in the mediation of cell death in p53 competent, TRAIL resistant cells occurs when co-treated with the gelsolin_1–70_ fragment.

## Discussion

We have previously identified that an N-terminal gelsolin fragment as a soluble mediator secreted from HCV core immortalized primary human hepatocytes mediates enhanced expression of the death receptors, TRAIL-R1 and TRAIL-R2, and induces apoptosis in LX2 cells^8,11^. Cytokine signaling through TNF receptors play a critical role in host defense by selectively eradicating infected and malignant cells from healthy cell populations^36^, and is hence of interest as a potential therapeutic agent. TRAIL-R1 and TRAIL–R2 facilitate the selective elimination of malignant cells through the induction of apoptosis via a caspase-dependent pathway^5^. A large number of tumorigenic cell lines suppress TRAIL mediated cell death using distinct mechanisms. Here, we identified that the N-terminal gelsolin fragment was able to synergistically aid TRAIL mediated cell death by modulating the MAPK/Akt and Bcl2/survivin signaling molecules. Previously, Huh7 and HepG2 cells exhibited minimal cell death in response to TRAIL or gelsolin^8,18,19^. To clarify the role of the N-terminal gelsolin sequence, a synthetic peptide comprising the amino acid residues 1–70 was utilized and was able to induce decreased cell proliferation in transformed hepatic cell lines treated concurrently with TRAIL.

We observed in this study that the gelsolin fragment or CM reduced the level of activated Akt with increased cell death. The activation of the MAPK/Erk pathway has been associated with the induction of Erk1/2 signal resulting in inhibition of caspase activation in response to TRAIL-R stimulation^37^. However, the addition of gelsolin containing CM suppressed activation of Erk in the presence of TRAIL. These results highlight the ability of gelsolin to stimulate TRAIL mediated induction of apoptosis by reducing the cells cyto-protective kinase pathways. Additionally, use of CM or the gelsolin fragment in the presence of TRAIL reduced the phosphorylation of Akt or ERK.

A number of studies have shown that treatment of tumor cells with chemotherapeutic agents and irradiation results in sensitization of TRAIL-resistant tumor cell lines^38–40^. However, the mechanisms leading to TRAIL sensitivity are controversial^38,41–43^. Here, we demonstrated that an N-terminal gelsolin fragment and TRAIL have a synergistic effect of inhibiting cell growth against TRAIL resistant transformed human hepatic cell lines. We observed that cell growth regulation by TRAIL and the gelsolin fragment are associated with multiple cellular mechanisms.

Sorafenib, a multi kinase inhibitor, can sensitize cancer resistant cells to cell death through the regulation of Stat3 and the downstream cytoprotective proteins, Mcl1 and survivin^44,45^. The use of sorafenib improves overall survival in patients with advanced HCC^17^. Activation of the Akt signaling pathway induces resistance to Sorafenib in hepatocellular carcinoma cells^46^. A co-culture of Huh7 with LX-2 cells induced phosphorylation of met, Akt, ERK and Stat3 associated with Sorafenib resistance^47^. The activation of the MAPK/Erk pathway associates with the induction of Erk1/2 resulting in inhibition of caspase activation in response to TRAIL-R stimulation^37^. These results highlight the ability of gelsolin to stimulate TRAIL mediated induction of apoptosis by reducing cyto-protective kinase pathways in cells, with the previously reported potential to eliminate activated stellate cells in culture.

We have observed that Akt and Bcl2 levels were significantly reduced with the concomitant treatment of Huh7 and HepG2 cells with the gelsolin fragment and TRAIL. Successful cancer treatment may be associated with understanding the cellular mechanisms that distinguish TRAIL-resistant from TRAIL-sensitive cells, and the ability to include treatment regimens that can overcome TRAIL resistance *in vivo*. HCC is one of the most common carcinomas, and options for successful therapy are limited due to chemotherapy resistance.

Targeting the stromal environment for cancer treatment is an emerging field. The Stromal environment constitutes the connective tissue framework of the liver and kidney distinguishing tumor from the tissue performing normal cell functions. We anticipate gelsolin will potentiate TRAIL mediated cell death in desmoplasia where resistant stromal cells are normally difficult to treat. Indeed, the LX2 model utilized by us was resistant to TRAIL mediated cell death, but exhibited decreased viability in the presence of the gelsolin fragment. The use of combination therapy focused upon the N-terminal gelsolin fragment and TRAIL could potentially be highly effective as a therapeutic agent together, or included in a treatment course with other therapeutics.

## Experimental procedures

### Cells

Immortalized human hepatocytes (IHH) were generated by transfection of a plasmid DNA expressing HCV core genomic region of genotype 1a (Genbank accession number M62321) into primary human hepatocytes under the control of a CMV promoter^48,49^, and cells were maintained in SAGM. Human transformed hepatocytes (Huh7, HepG2) were grown in DMEM, supplemented with 10% fetal bovine serum and antibiotics.

### Gelsolin

Purified gelsolin from human plasma was purchased (Sigma, MO). An N-terminal gelsolin peptide encompassing amino acids 1–70 was synthesized (Thermo Fisher), purified, and suspended in PBS. Aliquots were stored frozen at −70 °C. Peptide stock was diluted in cell culture medium for further use.

### Preparation of conditioned medium

IHH were grown on plastic plate at 37 °C as previously described^11^. Cells were washed and incubated in serum free medium for 48 h. CM was clarified by centrifugation at 6,000 g to remove cell debris, supplemented with 2X L-glutamine, and stored in aliquots at −20 °C until use.

### Cell proliferation assay

Cells were examined using a CellTiter 96 Aqueous One non-radioactive cell proliferation assay (Promega, WI), following the supplier’s protocol. Briefly, cells were plated on a 96 well format overnight, followed by exposure to IHH CM. After 24 hours, cells were exposed to the indicated concentration of TRAIL. Measurements were taken 48 hours after the addition of CM. In utilizing the gelsolin peptide, media was replaced 24 hours after plating with fresh medium in the presence or absence of peptide and TRAIL as above. LX2 cells were seeded at 50% confluency. On day 2, media was replaced with CM from IHH to 100 μl in each well, with serum free SAGM as a control. Cells were incubated at 37 °C for 48 hours. 50 μl aliquots from all test and control wells were transferred to a fresh 96-well flat clear bottom plate. 50 μl of the CytoTox 96® Reagent (Promega) was added to each sample aliquot, and incubated at room temperature for 30 min, and 50 μl of stop solution was added to each well. Absorbance was recorded at 490 nm. A maximum LDH release control was generated by adding 10 μl of 10X lysis solution (per 100 μl original volume) to the positive control wells 45 minutes before adding CytoTox 96® Reagent. Alternatively, an LDH release assay was performed using a 96-well assay plate. SC79 as an activator of Akt and siRNA to p53 were used in concert with the above treatment strategy to define the cellular mechanism of Gelsolin_1–70_/TRAIL mediated loss of cell viability.

### Western blot analysis

The status of and activation levels of select kinases and cell regulatory proteins were analyzed using specific monoclonal antibodies by Western blot analysis.

### Antibodies

Antibodies to TRAIL-R1/R2 (R&D Systems, MN), Bcl-2, Bax, p53 and Akt (Santa Cruz Biotechnologies, CA), phosho-Akt (S473), Survivin, phosho-Mdm2 (S166), phosphor-Erk (T202/Y204), p21, phospho-p53 (S15) (Cell Signaling, MA) and Actin (Sigma, MO) were procured and used in the study.
