# Author Correction: N-terminal gelsolin fragment potentiates TRAIL mediated death in resistant hepatoma cells

**DOI:** 10.1038/s41598-018-24095-7

**Published:** 2018-04-24

**Authors:** Keith Meyer, Young-Chan Kwon, Ratna B. Ray, Ranjit Ray

**Affiliations:** 1 Departments of Internal Medicine and Pathology, Saint Louis University, Missouri, USA; 2 Pathology, Saint Louis University, Missouri, USA

Correction to: *Scientific Reports* 10.1038/s41598-017-13131-7, published online 09 October 2017

In Figure 2D, the blot for ‘p-Erk (T202/Y204)’ is missing. The correct Figure 2 appears below as Figure 1.

**Figure 1:**
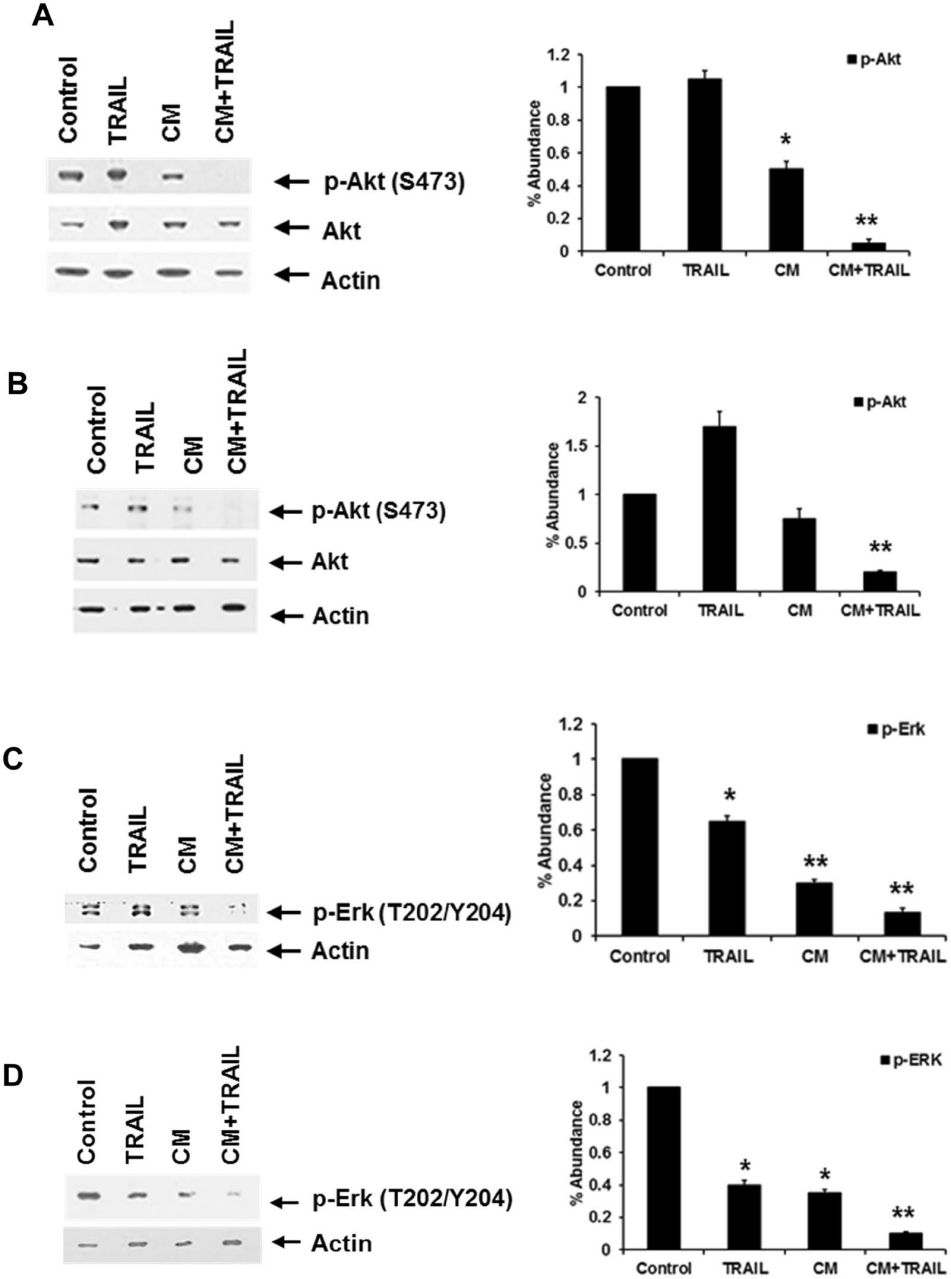
CM in association with TRAIL inhibits Akt and Erk activation. Akt and Erk activation was analyzed in HepG2 (panels A and C) and Huh7 (panels B and D) cells. CM was added on cells 24 hours prior to the addition of TRAIL. Densitometric scan data is provided. Each panel is representative of three independent experiments. Note that cropped gel images are used and the gels were run under the same experimental conditions.

